# Lung Cancer Stem Cell: New Insights on Experimental Models and Preclinical Data

**DOI:** 10.1155/2011/549181

**Published:** 2010-12-16

**Authors:** Caroline Rivera, Sofia Rivera, Yohann Loriot, Marie-Catherine Vozenin, Eric Deutsch

**Affiliations:** Laboratoire UPRES EA 27-10 “Radiosensibilité des Tumeurs et Tissus Sains”, Institut Gustave Roussy, 39 rue Camille Desmoulins, 94800 Villejuif, France

## Abstract

Lung cancer remains the leading cause of cancer death. Understanding lung tumors physiopathology should provide opportunity to prevent tumor development or/and improve their therapeutic management. Cancer stem cell (CSC) theory refers to a subpopulation of cancer cells, also named tumor-initiating cells, that can drive cancer development. Cells presenting these characteristics have been identified and isolated from lung cancer. Exploring cell markers and signaling pathways specific to lung CSCs may lead to progress in therapy and improve the prognosis of patients with lung cancer. Continuous efforts in developing *in vitro* and *in vivo* models may yield reliable tools to better understand CSC abilities and to test new therapeutic targets. Preclinical data on putative CSC targets are emerging by now. These preliminary studies are critical for the next generation of lung cancer therapies.

## 1. Introduction

Despite important improvements in therapies over the last decades, lung cancer prognosis remains very poor. After apparent successful initial therapy, development of secondary tumors often leads to a lethal relapse. Substantial growing experimental evidence has suggested that many cancers, including lung cancer, may be driven by a small subpopulation of self-renewing cells that could sustain malignant growth [1]. Innovative therapy could target this specific population of “cancer stem cells” (CSCs) or “tumor-initiating cells” in order to improve patients' outcome, with complete eradication of tumor growth. This review summarizes what is known about lung CSCs and focuses on potential clinical implications based on preclinical data obtained through new *in vitro* and *in vivo* models. 

## 2. Cancer Stem Cells

Two major models have been described for tumor propagation: the clonal evolution model, involving a stochastic component, and the CSC model defined as hierarchical. According to the clonal evolution model, neoplasm arise from a single cell of origin, and tumor progression results from acquired genetic variability within the original clone allowing sequential selection of more aggressive sublines [2]. CSC model sustains that tumor cells are heterogeneous and only the CSC subset has the ability to proliferate extensively and form new tumors [1, 3]. These models are not mutually exclusive [4]. Recent data provide support for the CSC hypothesis, which suggests that a relatively rare subpopulation of tumor cells have the unique ability to initiate and perpetuate tumor growth [5]. CSCs have been identified and isolated in various malignancies including solid tumors [6–10] such as lung cancer [11]. These cells can be defined as cancer cells that specifically possess the ability to give rise to all cell types found in a particular cancer sample. CSCs share various characteristics with embryonic and somatic stem cells including self-renewal and multipotent differentiation [12, 13]. Self-renewal is defined as the ability to go through unlimited cycles of cell division while maintaining the undifferentiated state. Stem cells are characterized by the capacity to renew themselves through mitotic cell division and differentiate into a diverse range of specialized cell types. Indeed, self-renewal, which drives tumorigenesis, and differentiation albeit aberrant that contributes to cellular heterogeneity of the tumor, are considered the key properties of the CSC.

Stem cell populations are established in niches, which are defined as specific locations that regulate how these cells participate in tissue regeneration, maintenance, and repair [14]. The stem cell niche represents the microenvironment that interacts with stem cells to regulate stem cell self-renewal and differentiation. The dependence of normal stem cells on the niche limits their expansion. CSCs might arise from normal stem cells that have acquired mutations that enable them to escape from niche control [15]. There is increasing evidence of the importance of the tumor and the host microenvironment in conditioning the stem cell status itself [16]. The expansion of the niche cells may be driven by alterations in the CSCs or in the niche cells themselves. In either case, there is an expansion of the self-renewing CSC pool that gives rise to aberrantly differentiated cancer cells, which comprise the bulk of the tumor [17]. Alterations in CSCs may enable them to commandeer alternative niche cells to provide them with self-renewal signals. Vascular niche in brain tumors has been shown to contribute directly to the generation of CSCs and tumor growth [18]. Although some stem cells are perivascular, others may occupy hypoxic niches and be regulated by O_2_ gradients [19]. However, the underlying mechanisms remain unclear. O_2_ availability may have a direct role in stem cell regulation through HIF-1*α* modulation of Wnt/*β*-catenin signaling [20]. If CSCs depend upon aberrant niche microenvironments, then these niches might represent targets for treatments.

Some fundamental characteristics are needed to recognize CSCs. The term CSC is frequently referred to as “cancer initiating cells”, since serial transplantation of CSCs into animal models re-establishes part of the phenotypic heterogeneity of the primary tumor [21]. Xenotransplantation in immunocompromised mice, followed by serial transplantation, is now regarded as an essential criterion in defining CSCs. This preponderant characteristic might represent the mechanistic parallel of regenerating a tissue or an organ in normal stem cell. These cells can be grown *in vitro* as tumor spheres under nonadherent conditions [22]. Self-renewal capacity also has to be documented.

CSCs have been shown in *in vitro* models to be highly resistant to radiation [23] and chemotherapy [24], potentially resulting in residual disease that can lead to recurrence. However, radioresistance of CSCs remains controversial. Inconsistent experimental results could be related to difficulty in correctly isolating the CSC subpopulation within a tumor [25]. CSCs may be less vulnerable to treatments because of intrinsic properties of radio and chemoresistance [26]. One hypothesis is that CSCs could be more resistant to DNA damages induced by these treatments [27, 28]. They may have enhanced capacities of DNA repair [29].

The frequency of CSCs appears to be highly variable, reflecting biological diversity among cancer models as well as technical issues. This subpopulation of cells was identified utilizing flow-cytometer-based cell sorting, enabling isolation of a “side population” (SP) defined by Hoechst 33342 dye exclusion [30]. This test is based on ABCG2 transporter, which is the second member of the G subfamily of ATP binding cassette (ABC) transporters. ABCG2 is one of the most important multidrug-resistance transporters and its substrates include Hoeschst 33342 [31]. This transporter is highly expressed in the SP containing stem cells [32]. CSCs are capable of self-renewal and production of differentiated progenitors. Progenitors are engaged in a differentiation pathway that progressively generates the different mature cells. CSCs express tissue-specific cell surface markers, such as CD34 in several kinds of leukemia [33] and CD44 in breast cancer [6]. None of these markers are specific to CSCs: CD34 is expressed by endothelial and hematologic normal cells, while CD44 is expressed by endothelial and mesenchymal cells. CD133 has been observed in a wide spectrum of malignant tumors including lung cancer [11, 34]. The function of CD133 is still unknown. CD133 may have a role in stem cell activation/maintenance [35], and potential regulatory activity of cell-cell contacts [36]. The CSC phenotype may not necessarily be uniform between cancer subtypes or even tumors of the same subtype [37]. Aldehyde dehydrogenase (ALDH) metabolizes a wide variety of endogenous and exogenous aldehydes [38]. High ALDH activity has been detected in stem and progenitor cells in various lineages [39–41], suggesting that strong ALDH activity and/or antigen expression can be used as a marker for CSC in a variety of cancers, including lung cancer [42].

## 3. Does the Cancer Stem Cell Concept Remain Disputed?

In the stochastic model, transformation results from a random mutation and subsequent clonal selection. But this model may not be sufficient to fully explain biology of carcinogenesis. The conceptual gap left by this model could be filled in by CSC hypothesis which becomes very attractive in this context. Indeed carcinogenesis involves acquisition and accumulation of genetic mutations and epigenetic regulations that may take many years. Since the normal lung epithelium turns over fairly rapidly, there must be some very long-lived cell type that serves as the repository to safeguard all these accumulating mutations. This can fit with CSC hypothesis and therefore explain both heterogeneity of cancer cells and events of metastasis. This theory may bring fundamental bases for understanding resistance to chemotherapy or radiation treatment, tumor promotion and progression, as well as important clinical implications for detection, prevention, and treatment [3]. However, some controversies still remain [43–45]. Tumor growth may not need to be driven by rare cancer stem cells. Cancer stem cell theory may not be an absolute requisite to explain chemo-radio resistance. This characteristic may be related to multiple drug resistance (mdr) phenotype [46]. Metabolic flexibility may generate adaptive changes in cells of the residual disease that can lead to tumor growth [47] and relapse. Tumor progression and recurrence may be explained by a state of cancer dormancy [48]. CSC may not be a real entity. CSC theory could be the simple transposition of normal tissue development to cancer. Although the CSC hypothesis still has to be fully validated in lung cancer, its applicability would provide an improvement in our understanding of carcinogenesis.

## 4. Lung Cancer

Lung cancer is one of the most intractable cancers with a five-year overall survival of around 15% all stages merged. Lung cancer has a poor prognosis, because of extra thoracic dissemination and frequent disease relapse. The main types of lung cancer are small cell lung carcinoma SCLC and nonsmall cell lung carcinoma NSCLC, which includes three major histological types: adenocarcinoma, squamous cell carcinoma (SCC), and large cell carcinoma [49]. About 15% of the tumors are SCLCs and arise in the larger airways, grow rapidly, and have a neuroendocrine component. Adenocarcinomas represent about 40% of NSCLCs and usually start in peripheral lung tissue. SCCs account for 25% and commonly originate near a central bronchus. Large cell carcinomas are believed to derive from neuroendocrine cells and may be observed in combination with other types of NSCLC. These neuroendocrine lung tumors appear to be epithelial tumors characterized by their preferential neuroendocrine differentiation as indicated by neuroendocrines granules, mucin granules, microvilli, and tonofilaments [50].

It is estimated that smoking causes 90% of lung cancers. However, different types of genetic modifications have been described in lung cancer cells such as recurrent chromosome abnormalities [51], upregulation of telomerase activity [52], and mutations of oncogenes or tumor suppressor genes (TP53, RB1, CDKN2A, KRAS, EGFR). Some may be markers of disease progression, others may have a direct role in lung cancer genesis in the context of gene-environment interactions [53]. The link between mutations and stem cell-ness is an important issue that needs to be explored. It is not clear whether mutations leading to CSCs occur in stem cells or in differentiated cells. These data strongly suggest that a deep knowledge of each tumor should improve patient outcome thanks to personalized therapy.

## 5. Normal Lung Stem Cells

The respiratory system arises from the ventral foregut endoderm with development of a tree-like system of epithelial tubules and vascular structures, which ultimately gives rise to the mature airways and alveoli. Precise sequence and pattern of branching events lead to highly ramified tubular networks possibly driven by a stereotyped hierarchical and modular branching program [54] which molecular regulation remains under study [55]. Lung mesenchymal development is crucially influenced by signals from the epithelium and the pleura that, in concert, appear to maintain a balance of differentiated and proliferating multipotent progenitors while the lung grows [56]. During lung branching morphogenesis, the transcription factor *Sox2* is associated with the epithelium that is less morphogenetically active. *Sox2 *expression is lost at sites where nascent buds arise [57, 58]. As epithelial cells in branching airways continue to differentiate, it is crucial to maintain and expand a pool of uncommitted progenitor cells for continuous growth. It has been proposed that this pool resides in the distal lung, as a population of proliferating immature epithelial cells that expresses high levels of the proto-oncogene *Mycn* [59].

**Figure 1:**
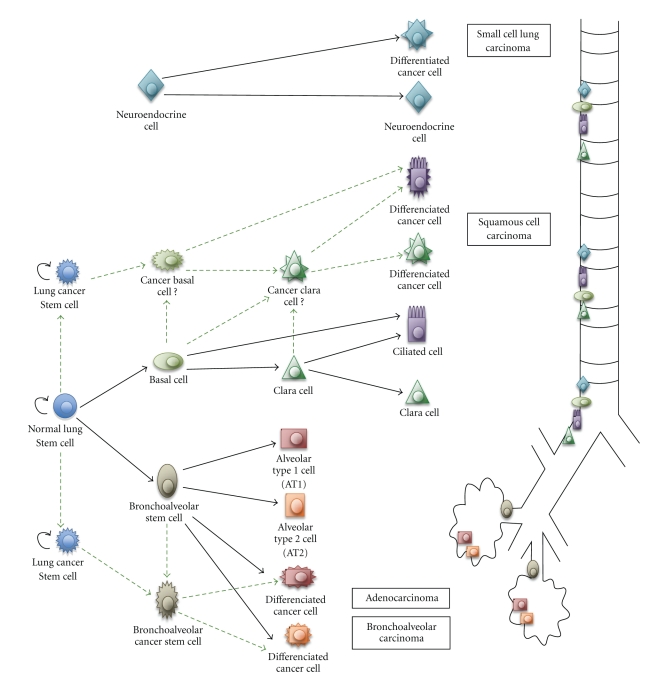
Lung CSCs and progenitors which differentiation leads to heterogeneity of associated human carcinomas. Normal and tumor cellular hierarchy progressively generates progenitor cells and differentiated mature cells in both proximal and distal airways. Hypotheses are represented with discontinuous lines.

Mature respiratory epithelium consists of multiple cell types. Ciliated, neuroendocrine, and secretory cells are located in the proximal region of the respiratory system. Alveolar type 1 (AT1) and 2 (AT2) cells are typical of the distal alveolar region of the lung (Figure 1). Lineage analysis suggests that progenitor cells lining the trachea and proximal lung have a different origin from those lining the distal region of the lung [60]. The activity of the different pools of progenitor cells account for spatially restricted regional specificities in both proximal and distal cell lineages specifications during lung development and cellular composition of tracheobronchial and bronchiolar airways [61]. Basal cells have been proposed as a multipotent progenitor cell population of bronchial airways. They have the capacity for restoration of a fully differentiated epithelium [62, 63]. Lung CSCs and airway normal stem cells may use common pathways in development.

## 6. Lung Cancer Stem Cells

Altered composition of airway epithelial cell that is associated with acute or chronic lung injury has the potential to significantly increase risk for developing lung cancer [64, 65]. It is currently unknown whether airway stem cells contribute significantly to normal epithelial maintenance and repair [66]. Clara cells participate in maintenance of both secretory and ciliated cell types after oxidant-mediated damage [67]. Meanwhile a population of pollutant-resistant stem cells localized in the bronchoalveolar duct junction (BADJ) contributes to restoration of a phenotypically diverse epithelium after clara cell depletion [68]. These cells are named bronchoalveolar stem cells (BASCs) (Figure 1). Both pulmonary neuroendocrine cells (PNEC) and Clara cells have been proposed as progenitors for the genesis of SCLC and NSCLC [69]. PNEC are specialized airway epithelial cells that produce specific neuropeptides and that are grouped in clusters termed neuroepithelial bodies (NEBs). Repair from airway injury is associated with PNEC hyperplasia [70]. Cells with Clara-like characteristics but reduced secretory protein expression have been identified in association with preneoplastic lesions [71]. These data suggest that cells engaged in differentiation could reactivate genes of immaturity. In this context, CSCs may arise from restricted progenitors or more differentiated cells that have reacquired self-renewing capacity [5]. This phenomenon could have direct bearing on the issue of stem cell plasticity, which is defined as the ability to cross lineage barriers and adopt cell-differentiated phenotypes [72]. It also may raise a parallel with transdifferentiation from differentiated cell to CSC that leads to cancer cell phenotypic diversification [73]. BASCs at the BADJ of adult airways have been proposed as an initiating cell source for lung adenocarcinoma [74]. Stem cells and progenitors could be considered ideal tumor initiating candidates, because dysregulation of proliferative capacity through mutation may rapidly cause dysplastic tumor-like growth. The deviation from a common endodermic stem cell could also be responsible for the multidirectional differentiation in lung tumors, including neuroendocrine phenotype in NSCLC and squamous or glandular signs of differentiation in authenticated neuroendocrine tumors [75].

Observed phenotypic heterogeneity between distinct tumor types suggests that the tumor's local pulmonary environment deeply impacts the fate of cancer cells [76]. Developmental programs of cells initially engaged in a specific lineage pathway can be altered by changing the type of signals in the local environment [77]. The different lung tumors could arise from regiospecific cancer stem cell niches that are still debated [78, 79].

## 7. Lung Cancer Stem Cell Models

**Figure 2:**
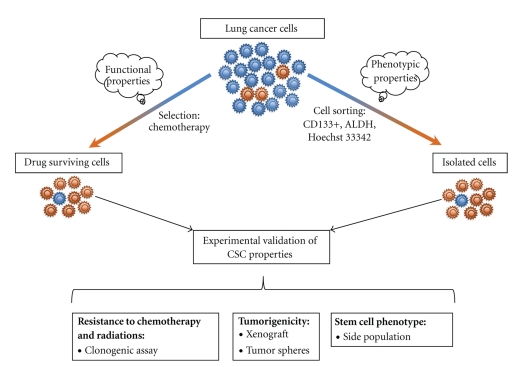
*In vitro* models of lung CSCs. Experimental models of lung CSCs are based on the functional properties of chemoresistance of these cells or on their phenotypic characteristics. Populations enriched in CSCs obtained after treatment or cell sorting need to present all the stemcellness properties to be considered as lung CSC models.

**Figure 3:**
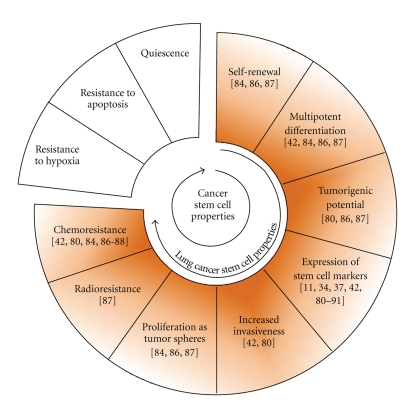
CSC and lung CSC properties. CSC characteristics verified on lung CSC models are presented with the references of the corresponding reports.

Tumorigenic human lung cancer cells have been isolated using different approaches from both cell lines and primary tumors. CSC models from lung cancer cell lines are based on the phenotype or on the functional characteristics of these cells (Figure 2). SP has showed repopulating ability and resistance to multiple chemotherapeutic drugs in lung cancer. In addition, human telomerase reverse transcriptase (hTERT) expression is higher in SP, suggesting that this fraction may represent an enriched source of lung tumor initiating cells with unlimited proliferative potential [80–83]. CSCs can be identified and isolated by flow-cytometry-based cell sorting using the cell surface marker CD133 [37] (Figure 2). The lack of early markers for lung progenitors represents a clear gap of knowledge. The known markers are not always ideal to sort for the CSC population [84]. In line with tumor heterogeneity, the phenotype of CSCs is not uniform, underlying the necessity to find more specific single markers or to define new marker combinations to potentially recognize the putative CSCs (Table 1). Isolation of lung CSCs based on increased ALDH activity was obtained using the Aldefluor assay followed by fluorescent-activated cell sorting (FACS) (Figure 2) [42, 85]. The second approach leading to the isolation of lung CSCs was based on their inherent functional resistance to chemotherapy (Figure 2). Drug-surviving cells were isolated after an *in vitro* treatment with cisplatin, doxorubicin, or etoposide. These cells present the phenotypic characteristics previously described [86]. These different CSC models developed on cancer cell lines can be grown *in vitro *as tumor spheres under nonadherent conditions using a serum-free medium that is supplemented with growth factors. They also exhibit high clonogenic potential, capacities for self-renewal, generation of differentiated progeny, and high *in vivo *tumorigenicity (Figure 3).

The first isolation and expansion of lung CSCs from primary patient tumors was based on their ability to survive under serum-free conditions and proliferate as cellular tumor spheres [11]. CD133 cells isolated from primary tumors displayed features of CSCs both *in vitro *and *in vivo *[87, 88]. The expression of CD133 in tumors was linked to shorter progression-free survival in patients treated with platinum-based chemotherapies, but this point remains controversial [88–91].

In vivo limiting dilution consists of injection of decreasing number of cells. This method used for subcutaneous tumor xenograft may prove enrichment of CSCs in an isolated subpopulation of cells [27, 92] (ratio to obtain a tumor from CSCs compared to tumor cells: 1/100). To date, there is no consistent data published on this method in lung CSC models that determine the minimal number of tumor-initiating cells necessary and sufficient to regenerate a tumor.

A few mouse models of lung cancer-initiating cells have been developed [74, 93]. However the frequency of CSCs in these models is highly variable. To date, there is no evidence of a subpopulation isolated from an animal model able to restore the tumor initial heterogeneity in secondary and tertiary hosts [84].

## 8. Preclinical Data on Lung Cancer Stem Cells

The surviving fraction of tumor cells after chemotherapy is low and may contain CSCs. These cells “left behind” may be responsible for recurrence. These cells need to be eradicated in order to provide long-term disease-free survival. Combinatorial treatments involving both cytotoxic and targeted therapies will probably be required to suppress all cancer cells [5]. Preclinical data on putative CSCs targets are emerging by now. 

Oncogenes and tumor-suppressor genes are the 2 types of genes involved in carcinogenesis. Oncogenes are dominant genes [94] whereas tumor suppressor genes are recessive. Activating mutations or transcriptional deregulation can lead from proto-oncogenes to oncogenes. The *Runx* genes can present characteristics of both oncogenes and tumor suppressor genes. These genes encode transcription factors involved in normal development with tissue specific expression. Many chromosomal translocations involving *Runx* genes and leading to oncogenic fusion proteins have been reported [95]. These fusion proteins may have a tissue-restricted oncogenic spectrum [96] in accordance with the concept of lineage-specific oncogenes. This concept applied to CSCs could suggest that some specific oncogenes could select and maintain a particular malignant phenotype characteristic of a cell lineage. *Runx3* is an essential transcription factor for the late phase of lung development. *Runx3* is required for the control of differentiation and proliferation of bronchiolar epithelium [97]. The frequent silencing of *Runx3* by promoter hypermethylation in the preneoplastic stage of the lung adenocarcinoma has been observed [98, 99]. *Runx3* downregulation has been proposed as an early event in the development of the lung carcinoma interfering in the differentiation of progenitor cells [97]. Moreover, there are some data suggesting that in lung cancer TITF1 could be an adenocarcinoma lineage specific oncogene [100] and *BRF2 *a squamous cell lineage specific oncogene [101].

BASC possesses the potential to differentiate into Clara or AT2 cells, presenting coexpression of Clara cell secretory protein (CCSP) and surfactant protein C (SPC) (Figure 1) [74]. These cells also exhibit the capacity of self-renewal and the expression of *Oct4*. *Oct4* is a transcriptional factor of the embryonic stem cells which expression was found to be associated with a poor prognosis [87, 102, 103]. In addition, knock down of *Oct4* might lead to apoptosis of a CSC-like population of lung cancer cells [104]. 

*Sox2 *is a critical transcription regulator of embryonic stem cells [105]. *Sox2* controls self-renewal and differentiation processes [106]. It is also implicated in lung branching morphogenesis during pulmonary development as previously evocated [57, 58]. *Sox2 *has been proposed to be an oncogene which expression is essential to lung SCC carcinogenesis and which is capable of transforming and conferring tumor initiating properties to human lung squamous cells [107]. Additionally, tumors with *Sox2 *overexpression seem to have a worse prognosis [108].

*Oct4 *and* Sox2* are two of the four crucial transcription factors capable of cooperating to reprogram differentiated cells into an induced pluripotent stem cell-like phenotype [109, 110]. Nuclear transplantation of *Oct4*, *Sox2*, *c-Myc*, and *Klf4* can reprogram a somatic genome back into an embryonic stem cell status. Is there a comparable way for cancer cells to reacquire an immature profile with properties of self-renewal? *Oct4* and *Sox2 *may participate to a similar process leading to lung CSCs.

Stem Cell Factor (SCF) is a mitogenic and angiogenic factor involved in carcinogenesis. Human *c-kit* has been shown to operate as a SCF receptor promoting tumor growth [111–113]. Patients with either mutation or overexpression of *c-kit* have lower survival rates and show resistance to chemotherapy [114]. Blocking SCF/*c-kit* signaling pathway inhibits CSC proliferation and survival after chemotherapy exposure in human lung cancer cell lines [115]. Exposure to SCF/*c-kit* axis blockade by SCF-neutralizing antibodies or by imatinib (Gleevec), an inhibitor of c-kit, combined to cisplatin therapy might lead to inhibition of both non-CSCs and CSCs.

Differences between normal stem cells and CSCs may provide novel antigenic and molecular targets for therapy (Table 1). It is important to design new therapeutic approaches to selectively hit CSC-specific pathways, while sparing normal stem cells. Further identification of lung cancer stem cell-specifics target could allow for promising more effective combined therapies. In our view, the most important matter to address in a near future is to better characterize and isolate lung CSCs from patients' tumors combining marker studies and functional assays. Lung CSCs probably include a heterogeneous population of cells with different therapeutic resistance profiles. Identifying lineage specific oncogenes or their fusion proteins could bring new targets for specific therapeutic approaches selectively aimed at specific CSCs. In contrast to chemotherapy or radiotherapy on mature tumor cells, therapies against CSCs might have a slow but sustainable effect. Therefore experimental models will certainly have to be redrawn including longer observation periods and intermediate analysis. 

## 9. Conclusion

CSC is central to cancer cell biology and cancer therapy. The knowledge of CSC signaling pathways may lead to new therapies able to eradicate lung CSC or induce differentiation of these cells. Promising models have recently been developed to isolate the subpopulation of CSCs within the lung-differentiated cells of the tumor mass. These models are useful tools to better characterize lung CSCs. Targeting the CSC or its microenvironmental niche could inhibit self-renewing and overcome chemo-radio resistance. Some consistent preclinical data are emerging on the role of transcriptional factors such as *Runx3*, *Oct4*, *Sox2*, and *c-kit *in lung tumor differentiation and treatment resistance. Further *in vitro *and* in vivo* studies are needed to design combined therapies in order to determine whether these candidates are potential targets for new therapeutic strategies against lung cancer. Moreover, the prognostic and predictive value of CSCs identification from tumor biopsies or among circulating tumor cells remains to be determined.

## Figures and Tables

**Table 1:** Molecular characterization of putative human lung CSCs.

Molecular characterization	References
Surface markers and transporters:	
CD133	[11, 34, 37, 87–91]
Side population—ABCG2	[80–83, 91]
ALDH activity	[42, 85]

Transcription factors:	
*Runx3*	[97–99]
*Sox2*	[107, 108]
*Oct4*	[87, 102–104]
*c-kit*	[111, 115]
